# Supplementary material for: Emergency department returns and early follow-up visits after heart failure hospitalization: Cohort study examining the role of race
Source: PLoS One. 2022 Dec 22;17(12):e0279394. doi: 10.1371/journal.pone.0279394 (PMC9778499; doi:10.1371/journal.pone.0279394)
Supplement: S1 File — (DOCX) [file pone.0279394.s001.docx]

Supporting Information:

**Explanatory Variable Operational Definitions**

**7-day scheduled follow-up:** Early follow-up was defied as having a scheduled appointment within seven days post-discharge with any outpatient provider.

**Patient received other transition of care interventions:** Two or three ‘other’ interventions are determined at the cluster level based on local gaps in the transition of care process collaboratively identified by the cluster team; patients who received one or more of these other I-MPACT intervention(s) are coded 1 and others 0.

**Race:** White/Black or African American

**Age:** was defined as under 75 years/75 years or over;

**Married:** Marital status was assessed using categories married, single, separated, widowed and divorced; for analysis, married patients were coded 1 and single, separated, widowed and divorced patients were coded 0;

**Neighborhood income**: Zip code median household income from the 2006-2010 American Community Survey (<https://www.psc.isr.umich.edu/dis/census/Features/tract2zip/>), and was measured in units of $1000 for purposes of analysis .

**Medicaid**: Abstracted patient medical insurance types included: BCBS Michigan ,BCN Michigan, Commercial - HMO ,Medicaid - HMO ,Medicaid - Straight ,Medicare - Original ,Medicare Advantage - BCBSM ,Medicare Advantage - BCN ,Medicare Advantage (Non-BCBSM / Non-BCN), No Insurance / Self Pay ,Other Payer - Government ,Other Payer - Michigan and Out-of-State, and Program of All-Inclusive Care for the Elderly (PACE). Patients with Medicaid were coded 1 and others were coded 0.

**Charlson:** Comorbidity Index with scores ranging from 1-16 among patients

**Length of stay (LOS):** Measured in days and categorized as 0-2, 3-4, 5-7, >7

**ED visits in prior 180 days:** The number of emergency department visits the patient has had in the 180 days prior to the index admission was measured with 0, 1, 2 , 3, 3 or more, and dichotomized for this study combining 1 or more visits.

Admitted from Emergency Department: Patient's admission source for the index admission was assessed using categories emergency department, operating room, direct admission from outpatient setting, hospital transfer, observation unit/status, and other, and emergency department was coded 1 and remaining categories together were coded 0.

**Treated in Intensive Care Unit during admission**: Patients treated in the ICU at some point during the index hospitalization were coded 1 and others coded 0.

**Admissions in prior 180 days**: Unplanned hospitalizations the patient had within the 180 days prior to the index admission was assessed with 0, 1, 2 , 3, 3 or more, and dichotomized for this study combining 1 or more hospitalizations.

**PCP identified in Discharge Summary:** Patients with an identified primary care physician (PCP) in the hospital discharge summary or patients referred to a clinic to obtain a PCP were coded 1 while others were coded 0.

**Discharged with Opioids:** Patients discharged on Opioids/Narcotics were coded 1 while others were coded 0.

**Discharged with Antiplatelets:** Patients discharged on anti-platelet medications (including Anagrelide (Agrylin), Aspirin, Aspirin/extended release dipyridamole (Aggrenox), Aspirin extended-release (Durlaza), Cilostazol (Pletal), Clopidogrel (Plavix), Dipyridamole (Persantine), Prasugrel (Effient), Ticlopidine, Vorapaxar (Zontivity), Other anti-platelets not mentioned above) were coded 1 and others coded 0

**Discharged with ≥10 medications:** Assessed using the question **‘**Based on information in the hospital discharge summary, how many medications was the patient discharged with’ and categories 0-1, 2-5, 6-10, >10.

DS / AVS medication discrepancy: This assessed whether there were discrepancies in the patient's listed medications between the After Visit Summary and the Discharge Summary.

**Depression:** Patients for whom depression was listed as a diagnosis in patient's hospital discharge summary or prescribed anti-depressants at discharge according to the hospital discharge summary were considered as having depression.

**Uncontrolled Diabetes:** According to the patient's medical record, if the patient’s diabetes was uncontrolled or if the patient's most recent A1C was above eight, the patient was considered as having uncontrolled diabetes.

**Dialysis:** Moderate or severe renal disease is documented as a condition in the patient's medical record and ‘patient requires dialysis’ is noted in the medical record regarding how the patient is currently managing their renal disease. Other methods of management listed in survey were: patient has had a prior renal transplant, patient does not require dialysis and has not had a renal transplant, or unknown
