# Supplementary material for: Emergency department returns and early follow-up visits after heart failure hospitalization: Cohort study examining the role of race
Source: PLoS One. 2022 Dec 22;17(12):e0279394. doi: 10.1371/journal.pone.0279394 (PMC9778499; doi:10.1371/journal.pone.0279394)
Supplement: S1 Table — P-values for categorical patient measures are based on chi-square test, while p-values for continuous measures are based on linear regression. a Neighborhood income is zip code median household income measured in units of $1K. b Charlson Comorbidity Index (1–12) c Abbreviations: ED, Emergency Department; PCP, Primary Care Provider; AVS, After Visit Summary; DS, Discharge Summary. d Other quality improvement transitional care intervention(s): intervention(s) determined at the cluster level excluding 7-day follow-up, activities such as care management, standardized discharge process, consults/referrals, patient education, or medication reconciliation. (DOCX) [file pone.0279394.s002.docx]

| **S1 Table. Patient Characteristics Stratified by Race** | | | | | | | | |
| --- | --- | --- | --- | --- | --- | --- | --- | --- |
|  | **Black^a^** | | | | **White^a^** | | | |
|  | **Overall** | **Early Follow-up** | **No Early Follow-up** | **p-value** | **Overall** | **Early Follow-up** | **No Early Follow-up** | **p-value** |
|  | **(N=2,401)** | **(N=863)** | **(N=1,538)** |  | **(N=4,092)** | **(N=1,935)** | **(N=2,157)** |  |
| **Sociodemographics** |  |  |  |  |  |  |  |  |
| Age (SD) | 63.8 (14.6) | 64.6 (14.5) | 63.4 (14.6) | 0.048 | 75.3 (12.6) | 75.3 (12.3) | 75.2 (12.9) | 0.908 |
| Female | 50.0% | 49.9% | 50.0% | 0.978 | 50.1% | 48.8% | 51.3% | 0.113 |
| Married | 29.3% | 31.5% | 28.1% | 0.077 | 47.6% | 52.0% | 43.6% | <0.001 |
| Medicaid | 17.4% | 14.6% | 19.0% | 0.007 | 3.6% | 3.2% | 4.1% | 0.114 |
| Neighborhood Income ($1000) (SD)^a^ | 39.9 (16.5) | 40.3 (16.6) | 39.6 (16.5) | 0.311 | 59.1 (19.6) | 57.9 (18.4) | 60.2 (20.6) | 0.0001 |
| **Clinical Characteristics: Patient** |  |  |  |  |  |  |  |  |
| Charlson Comorbidity Index (SD)^b^ | 4.2 (2.1) | 4.3 (2.1) | 4.1 (2.1) | 0.009 | 4.2 (2.2) | 4.3 (2.2) | 4.1 (2.2) | 0.001 |
| Diabetes uncontrolled | 10.4% | 10.8% | 10.1% | 0.625 | 8.0% | 9.4% | 6.9% | 0.003 |
| Discharged with ≥10 medications | 60.3% | 64.4% | 58.0% | 0.002 | 70.3% | 73.1% | 67.7% | <0.001 |
| Discharged with Opioids | 25.3% | 24.9% | 25.6% | 0.729 | 23.9% | 22.9% | 24.7% | 0.174 |
| Discharged on Antiplatelets | 65.7% | 63.4% | 67.0% | 0.070 | 61.0% | 59.7% | 62.2% | 0.112 |
| Depression | 20.7% | 20.9% | 20.6% | 0.886 | 31.0% | 30.7% | 31.3% | 0.658 |
| Required Dialysis | 13.8% | 13.4% | 14.0% | 0.714 | 5.5% | 4.0% | 7.0% | <0.001 |
| **Clinical Characteristics: Hospitalization** |  |  |  |  |  |  |  |  |
| ED visits within 30 days | 12.2% | 10.8% | 13.1% | 0.100 | 9.1% | 8.7% | 9.4% | 0.452 |
| ED visits in prior 180 days | 38.2% | 39.2% | 37.7% | 0.482 | 28.3% | 29.5% | 27.3% | 0.127 |
| Admissions in prior 180 days | 52.4% | 51.6% | 52.9% | 0.522 | 46.9% | 45.8% | 47.9% | 0.189 |
| Treated in Intensive Care Unit | 8.5% | 9.0% | 8.3% | 0.511 | 7.4% | 7.4% | 7.5% | 0.928 |
| PCP identified in Discharge Summary ^c^ | 84.8% | 89.0% | 82.4% | <0.001 | 86.4% | 90.3% | 83.0% | <0.001 |
| DS/AVS medication discrepancy ^c^ | 58.9% | 49.2% | 64.2% | <0.001 | 48.8% | 43.8% | 53.3% | <0.001 |
| Length of Stay (SD) ^c^ | 4.6 (3.4) | 4.6 (3.1) | 4.6 (3.5) | 0.789 | 4.9 (3.8) | 4.8 (3.4) | 4.9 (4.0) | 0.847 |
| Received other transitional care/s^d^ | 70.7% | 78.6% | 66.3% | <0.001 | 74.5% | 83.1% | 66.8% | <0.001 |
| Admitted from ED ^c^ | 90.3% | 89.5% | 90.7% | 0.323 | 89.6% | 87.3% | 91.6% | <0.001 |

P-values for categorical patient measures are based on chi-square test, while p-values for continuous measures are based on linear regression (a) Neighborhood income is zip code median household income measured in units of $1K (b) Charlson Comorbidity Index (1-12) (c) Abbreviations: ED, Emergency Department; PCP, Primary Care Provider; AVS, After Visit Summary; DS, Discharge Summary (d) Other quality improvement transitional care intervention(s) intervention(s) determined at the cluster level excluding 7 day follow up, activities such as care management, standardized discharge process, consults/ referrals, patient education, or medication reconciliation.
