# Supplementary material for: Emergency department returns and early follow-up visits after heart failure hospitalization: Cohort study examining the role of race
Source: PLoS One. 2022 Dec 22;17(12):e0279394. doi: 10.1371/journal.pone.0279394 (PMC9778499; doi:10.1371/journal.pone.0279394)
Supplement: S2 Table — (a) Comparison is for 10th and 90th percentile values. (b) Abbreviations: ED, Emergency Department; PCP, Primary Care Provider; AVS, After Visit Summary; DS, Discharge Summary (c) Other quality improvement transitional care intervention(s): intervention(s) determined at the cluster level excluding 7-day follow-up, activities such as care management, standardized discharge process, consults/referrals, patient education, or medication reconciliation. (DOCX) [file pone.0279394.s003.docx]

| **S2 Table. Unadjusted Associations Between Early Follow-up and Emergency Department Return Visits** | | | |
| --- | --- | --- | --- |
|  | **Risk Ratio** | **95% CI** | **p-value** |
| **Variable of Interest** |  |  |  |
| Early Follow-up | 0.86 | [0.73, 0.99] | 0.039 |
| **Sociodemographics** |  |  |  |
| Age >75 Years | 0.81 | [0.69, 0.93] | 0.005 |
| Female | 0.93 | [0.80, 1.07] | 0.344 |
| Black | 1.35 | [1.16, 1.55] | <0.001 |
| Married | 0.85 | [0.73, 0.98] | 0.032 |
| Medicaid | 1.47 | [1.18, 1.81] | 0.001 |
| Neighborhood Income ($10,000)^a^ | 0.90 | [0.82, 0.98] | 0.028 |
| **Clinical Characteristics: Patient** |  |  |  |
| Charlson Comorbidity Index ^a^ | 1.43 | [1.22, 1.68] | <0.001 |
| Diabetes uncontrolled | 1.16 | [0.91, 1.47] | 0.208 |
| Discharged with ≥10 medications | 1.19 | [1.01, 1.38] | 0.034 |
| Discharged with Opioids | 1.31 | [1.12, 1.53] | 0.001 |
| Discharged on Antiplatelets | 1.16 | [0.99, 1.35] | 0.052 |
| Depression | 1.22 | [1.04, 1.42] | 0.011 |
| Required Dialysis | 2.13 | [1.76, 2.55] | <0.001 |
| **Clinical Characteristics: Hospitalization** |  |  |  |
| ED visits in prior 180 days ^b^ | 2.15 | [1.86, 2.48] | <0.001 |
| Admissions in prior 180 days | 1.78 | [1.53, 2.06] | <0.001 |
| Treated in Intensive Care Unit | 0.91 | [0.69, 1.20] | 0.524 |
| PCP identified in Discharge Summary ^b^ | 1.01 | [0.81, 1.23] | 0.956 |
| DS/AVS medication discrepancy ^b^ | 1.02 | [0.88, 1.17] | 0.798 |
| Length of Stay ^a,b^ | 0.91 | [0.79, 1.04] | 0.18 |
| Received other transitional care/s ^c^ | 0.92 | [0.78, 1.07] | 0.272 |
| Admitted from ED ^b^ | 1.16 | [0.90, 1.50] | 0.243 |
| (a) Comparison is for 10th and 90th percentile values. (b) Abbreviations: ED, Emergency Department; PCP, Primary Care Provider; AVS, After Visit Summary; DS, Discharge Summary (c) Other quality improvement transitional care intervention(s) intervention(s) determined at the cluster level excluding 7 day follow up, activities such as care management, standardized discharge process, consults/ referrals, patient education, or medication reconciliation. | | | |
