# Supplementary material for: Emergency department returns and early follow-up visits after heart failure hospitalization: Cohort study examining the role of race
Source: PLoS One. 2022 Dec 22;17(12):e0279394. doi: 10.1371/journal.pone.0279394 (PMC9778499; doi:10.1371/journal.pone.0279394)
Supplement: S3 Table — Adjusted Risk Ratios (aRRs) from multivariable logistic regression model between early follow-up (scheduled within 7 days post-discharge) and patient/site factors. Panel A shows model with Black race variable and no site interaction term. Panel B shows model that included Panel A variables with an additional race x site interaction term. We present the predicted adjusted association between Black race and 7-day follow-up by each hospital site ordered by percentage of Black patients hospitalized for heart failure in each site. (DOCX) [file pone.0279394.s004.docx]

| **S3 Table. Adjusted Association between Race and Early Follow-up by Aggregate and by Hospital Site in Black and White Patients Hospitalized for Heart Failure at 13 Hospitals in Michigan From 2017 to 2020.** | | |
| --- | --- | --- |
| **Panel A: Adjusted Association of Patient Characteristics and Early Follow-up** | | |
| **Variables** | **ARR (95% CI)** | **P Value** |
| **Sociodemographics** |  |  |
| Black | 0.95 (0.89-1.01) | 0.12 |
| Married | 1.12 (1.06-1.18) | <0.001 |
| Medicaid | 0.90 (0.80-1.00) | 0.04 |
| **Clinical Characteristics: Patient** |  |  |
| Charlson Index (p90=7 vs.p10=2) | 1.01 (0.95-1.08) | 0.72 |
| Discharged with ≥10 medications | 1.12 (1.06-1.20) | 0.00 |
| Discharged with Opioids | 0.94 (0.88-1.00) | 0.05 |
| Depression | 0.92 (0.86-0.98) | 0.01 |
| Dialysis | 0.86 (0.77-0.96) | 0.01 |
| **Clinical Characteristics: Hospital** |  |  |
| ED Visits in prior 180 days | 1.03 (0.97-1.09) | 0.32 |
| Admission/s in prior 180 days | 0.97 (0.92-1.02) | 0.24 |
| PCP Identified in Discharge Summary | 1.35 (1.23-1.48) | <0.001 |
| LOS (p90=8 days vs.p10=2 days) | 0.99 (0.95-1.04) | 0.70 |
| Received other transitional care/s | 1.36 (1.26-1.46) | <0.001 |
| Admitted from ED | 0.92 (0.84-1.00) | 0.05 |
| **Panel B: Adjusted Association of Black Race and Early Follow-up by Hospital** | | |
| **Site** | **ARR (95% CI)** | **P Value** |
| site 1 (Black=9%) | 0.84 (0.59-1.19) | 0.36 |
| site 2 (Black=10%) | 0.45 (0.21-0.96) | 0.04 |
| site 3 (Black=15%) | 0.66 (0.43-1.00) | 0.05 |
| site 4 (Black=19%) | 0.85 (0.72-0.99) | 0.04 |
| site 5 (Black=24%) | 0.88 (0.74-1.03) | 0.15 |
| site 6 (Black=26%) | 1.10 (0.92-1.32) | 0.35 |
| site 7 (Black=28%) | 0.94 (0.81-1.10) | 0.41 |
| site 8 (Black=30%) | 1.19 (0.98-1.43) | 0.08 |
| site 9 (Black=30%) | 0.91 (0.62-1.36) | 0.49 |
| site 10 (Black=44%) | 1.42 (0.96-2.10) | 0.08 |
| site 11 (Black=65%) | 1.00 (0.74-1.35) | 0.94 |
| site 12 (Black=78%) | 0.98 (0.79-1.22) | 0.95 |
| site 13 (Black=92%) | 1.08 (0.63-1.83) | 0.80 |
| *Notes****.*** *Adjusted Risk Ratios (aRRs) from multivariable logistic regression model between early follow-up (scheduled within 7 days post-discharge) and patient/site factors. Panel A shows model with Black race variable and no site interaction term. Panel B shows model that included Panel A variables with an additional race x site interaction term. We present the predicted adjusted association between Black race and 7-day follow-up by each hospital site ordered by percentage of Black patients hospitalized for heart failure in each site.* | | |
