# Supplementary material for: Emergency department returns and early follow-up visits after heart failure hospitalization: Cohort study examining the role of race
Source: PLoS One. 2022 Dec 22;17(12):e0279394. doi: 10.1371/journal.pone.0279394 (PMC9778499; doi:10.1371/journal.pone.0279394)
Supplement: S4 Table — (a) Comparison is for 10th and 90th percentile values. (b) Abbreviations: ED, Emergency Department; PCP, Primary Care Provider; AVS, After Visit Summary; DS, Discharge Summary (c) Other quality improvement transitional care intervention(s): intervention(s) determined at the cluster level excluding 7-day follow-up, activities such as care management, standardized discharge process, consults/referrals, patient education, or medication reconciliation. (DOCX) [file pone.0279394.s005.docx]

| **S4 Table. Unadjusted Associations Between Patient Characteristics and Early Follow-up** | | | |
| --- | --- | --- | --- |
|  | **Risk Ratio** | **95% CI** | **p-value** |
| **Variable of Interest** |  |  |  |
| Black | 0.76 | [0.71, 0.80] | <0.001 |
| **Sociodemographics** |  |  |  |
| Age >75 Years | 1.11 | [1.05, 1.17] | <0.001 |
| Female | 0.96 | [0.91, 1.01] | <0.001 |
| Married | 1.22 | [1.15, 1.29] | <0.001 |
| Medicaid | 0.75 | [0.66, 0.84] | <0.001 |
| Neighborhood Income ($10,000)^a^ | 1.03 | [0.99, 1.06] | 0.115 |
| **Clinical Characteristics: Patient** |  |  |  |
| Charlson Comorbidity Index ^a^ | 1.16 | [1.08, 1.23] | <0.001 |
| Diabetes uncontrolled | 1.11 | [1.01, 1.21] | 0.023 |
| Discharged with ≥10 medications | 1.20 | [1.12, 1.27] | <0.001 |
| Discharged with Opioids | 0.95 | [0.89, 1.01] | 0.155 |
| Discharged on Antiplatelets | 0.92 | [0.87, 0.97] | 0.006 |
| Depression | 1.02 | [0.96, 1.08] | 0.478 |
| Required Dialysis | 0.79 | [0.70, 0.88] | <0.001 |
| **Clinical Characteristics: Hospitalization** |  |  |  |
| ED visits in prior 180 days ^b^ | 1.02 | [0.96, 1.08] | 0.485 |
| Admissions in prior 180 days | 0.95 | [0.89, 1.01] | 0.058 |
| Treated in Intensive Care Unit | 1.01 | [0.90, 1.11] | 0.877 |
| PCP identified in Discharge Summary ^b^ | 1.47 | [1.32, 1.62] | <0.001 |
| DS/AVS medication discrepancy ^b^ | 0.75 | [0.71, 0.79] | <0.001 |
| Length of Stay ^a,b^ | 1.01 | [0.96, 1.05] | 0.786 |
| Admitted from ED ^b^ | 0.83 | [0.76, 0.89] | <0.001 |
| (a) Comparison is for 10th and 90th percentile values. (b) Abbreviations: ED, Emergency Department; PCP, Primary Care Provider; AVS, After Visit Summary; DS, Discharge Summary (c) Other quality improvement transitional care intervention(s): intervention(s) determined at the cluster level excluding 7-day follow-up, activities such as care management, standardized discharge process, consults/referrals, patient education, or medication reconciliation. | | | |
