# Supplementary material for: Emergency department returns and early follow-up visits after heart failure hospitalization: Cohort study examining the role of race
Source: PLoS One. 2022 Dec 22;17(12):e0279394. doi: 10.1371/journal.pone.0279394 (PMC9778499; doi:10.1371/journal.pone.0279394)
Supplement: Attachment — Submitted filename: Response to Reviewers_080822_RS.docx [file pone.0279394.s006.docx]

Reviewers' comments:

Reviewer's Responses to Questions

**Comments to the Author**

1. Is the manuscript technically sound, and do the data support the conclusions?

The manuscript must describe a technically sound piece of scientific research with data that supports the conclusions. Experiments must have been conducted rigorously, with appropriate controls, replication, and sample sizes. The conclusions must be drawn appropriately based on the data presented.

Reviewer #1: Partly

Reviewer #2: Yes

2. Has the statistical analysis been performed appropriately and rigorously?

Reviewer #1: I Don't Know

Reviewer #2: Yes

3. Have the authors made all data underlying the findings in their manuscript fully available?

The [PLOS Data policy](http://www.plosone.org/static/policies.action#sharing) requires authors to make all data underlying the findings described in their manuscript fully available without restriction, with rare exception (please refer to the Data Availability Statement in the manuscript PDF file). The data should be provided as part of the manuscript or its supporting information, or deposited to a public repository. For example, in addition to summary statistics, the data points behind means, medians and variance measures should be available. If there are restrictions on publicly sharing data—e.g. participant privacy or use of data from a third party—those must be specified.

Reviewer #1: No

Reviewer #2: No

4. Is the manuscript presented in an intelligible fashion and written in standard English?

PLOS ONE does not copyedit accepted manuscripts, so the language in submitted articles must be clear, correct, and unambiguous. Any typographical or grammatical errors should be corrected at revision, so please note any specific errors here.

Reviewer #1: Yes

Reviewer #2: Yes

**5. Review Comments to the Author**

Please use the space provided to explain your answers to the questions above. You may also include additional comments for the author, including concerns about dual publication, research ethics, or publication ethics. (Please upload your review as an attachment if it exceeds 20,000 characters)

Reviewer #1: Summary: Solnick et al. has submitted a study in which they examine if Black race and other patient factors are associated with receiving a scheduled 7-day follow-up after HF hospitalization discharge and whether early follow- up is associated with lower 30-day ED returns. They conducted a retrospective cohort analysis of 6493 Black and White patients at thirteen Michigan hospitals. They reported that early follow-up visits were associated with a lower likelihood of ED returns for HD patients.

INTRODUCTION

1. It is unclear what the author means by “worse outcomes”. What types of outcomes occur in these patients.

This has been clarified: *Black patients face the worst heart disease survival rates of all racial groups[1] and in particular, black patients experience higher age-adjusted heart failure (HF) death rates compared to White patients.[*

2. Please explain what “transitional care” is.

This is explained now as: “*Defined as services a patient receives when changing healthcare settings and including telephone follow-ups or early follow-up visits, transitional care,”*

3. It is unclear if there is any literature on early follow up visits and reduced ED times. If so, please cite existing literature. If not, please note that there is no current literature on the matter.

I am unsure of what is meant by “reduced ED times.” Does this mean ED length of stay? If so, there is not literature on this that I am aware of. However, if this is referring to ED visits there is a meta analysis by Vedel et al on the impact of transitional care (varying between telephone calls/ clinic follow-up/ structure home visit ) a 29% overall reduction in ED visits after HR admission. The only one that included clinic follow up was the Duchame paper which did not have a significant effect. The significant studies below were Tsukyiki and Harrison which were a telephone follow-up and structured home visits combined with telephone follow-up (The forest plot for above meta-analysis is copied below for reference).

4. Please clarify why the authors specifically are examining Black race.

We are examining Black race due to evidence this group has the highest rates of heart failure and evidence there are implicit biases that drive the type of heart failure care they receive. Thus, it may be that they also receive less transitional care and if so, the reason for less transitional care may be bias. The rationale is now in the introduction.

5. It is not enough to say “additional patient factors” in the objective sentence. Please clarify specific factors that the authors are interested in.

This has been revised as such: “We examine whether Black race, Medicaid, and certain clinical factors were associated with receiving early follow-up after HF hospitalization and whether early follow-up is associated with lower ED returns visits within 30 days.”

The rationale for choosing comorbidities and Medicaid follows similar logic as with selecting Black race, low-income patients and those with comorbidities have higher healthcare utilization for HF, thus if targeted with early follow-ups may have reduced ED return and hospital readmission.

6. Please note the hypothesis for early follow up and 30-day ED returns

This has been added to the Intro “We hypothesized that Black and Medicaid patients are less likely to have an early follow-up scheduled, and that early follow-up is associated with lower ED returns”

7. Please provide background information on why it is important to study seven follow ups.

Additional citations including these have been included:

Lee, K. K., Yang, J., Hernandez, A. F., Steimle, A. E., & Go, A. S. (2016). Post-discharge follow-up characteristics associated with 30-day readmission after heart failure hospitalization. Medical care, 54(4), 365.

11,985 HF patients; Used logistic regression. Outpatient contact within 1-7 days after discharge was associated with lower odds of readmission (adjusted odds ratio [OR] 0.81, 95% CI: 0.70–0.94), whereas later outpatient contact between 8 and 30 days after hospital discharge was not significantly associated with readmission (adjusted OR 0.99, 95% CI: 0.82–1.19).

Tung, Y. C., Chang, G. M., Chang, H. Y., & Yu, T. H. (2017). Relationship between early physician follow-up and 30-day readmission after acute myocardial infarction and heart failure. PLoS One, 12(1), e0170061.

The study population for non-ST-segment-elevation myocardial infarction (NSTEMI) and heart failure included 5,008 and 13,577 patients, respectively. Used Cox regression. A completed 7d follow-up was associated with a lower hazard ratio of readmission compared with no early physician follow-up for patients with NSTEMI (hazard ratio [HR], 0.47; 95% confidence interval [CI], 0.39±0.57), and for patients with heart failure (HR, 0.54; 95% CI, 0.48±0.60). They found similar results for a completed 0-14d f/u.

These studies above are cited in this sentence in the introduction “Defined as services a patient receives when changing healthcare settings and including telephone follow-ups or early follow-up visits, transitional care, and specifically 7-day follow-up visits, have been associated with reduced hospital readmissions following HF hospitalization. [16–18]”

8. Please place the study within the context of Michigan and why this study is being conducted in this location.

As for the justification regarding the 13 hospitals:

Hospitals throughout the state of Michigan were recruited, participation was voluntary. Once a hospital agreed to participate, they were given a choice of populations to work with, CHF, COPD or patients transitioning into and out of a SNF.

Context:

The study was conducted in Michigan as Blue Cross Blue Shield of Michigan provides the funding for I-MPACT and other quality collaboratives.

This is described in the Introduction: “We conduct this study in a quality improvement collaborative of 13 hospitals across Michigan which provide data on a set of specific targeted conditions in which HF is one. Hospitals across Michigan were recruited, participation was voluntary. This unique multi-payer database allows for examination of care across a variety of hospital settings with all-payer patient data for HF….. “. Charts were manually abstracted by trained chart abstractors as part of a quality improvement initiative funded by Blue Cross Blue Shield of Michigan (BCBSM) and Blue Care Network

METHODS

1. It is unclear how the authors categorized Black and White. How were mixed race individuals categorized for this study? Furthermore, please clarify how Hispanic individuals were categorized. Finally, please provide justification as to why only Black and White individuals were included in the study.

Only Black and White individuals were included because of prior research identifying the largest racial disparities in HF outcomes between these groups- this is now discussed more thoroughly in the introduction. Patient race was assessed using categories, American Indian or Alaska Native, Asian, Black or African American, Native Hawaiian or Other Pacific Islander, White or Caucasian, and Other Race. For patients who chose, ‘Other Race’ a follow-up question allowed text fields to describe their racial identification, and only one patient identified as mixed race, i.e. ‘Black/African American or White’. The initial sample (N=6,613) was 61.86% White or Caucasian, 36.31% Black or African American while 121 (1.83%) patients belonged to some other race. In the initial sample 45 (0.68%) patients self-identified as Hispanic, 93.35% as Non-Hispanic, and ethnicity data was missing for 395 (5.97%) patients. Due to low number, 121 patients who identified as ‘some other race’ were excluded from analysis. Due to low variation and high missing data in ethnicity, ethnicity could not be considered as a covariate.

2. Please provide justification as to why the thirteen hospitals were chosen. Additionally, please provide details on these hospitals such as location (urban vs rural), type (public vs private), demographics (race).

Details on urban vs rural was unavailable, but is available for designation of critical access hospital, of which this group includes none. Methods now state: “Details of the involved hospitals are as follows: all non-profit and general acute care hospitals, 6 church ownership, 7 private ownership, 12 teaching hospitals, hospital beds range from 129-637 beds, 0 critical access designated, and distribution of patient race is provided in Figure 1.”

3. Was race the only variable that was self reported?

All data, including race, was abstracted from the medical record. Race was self-reported in the medical record.

4. The author wrote “Early follow-up visits were incentivized by BCBSM through pay-for-performance and were counted if scheduled”. It is unclear if all patients received this incentive or only those that had BCBSM insurance. If the latter, this should be taken into account in the analysis.

Hospitals were incentivized through pay-for-performance to increase scheduling of post-discharge follow-up visits for patients. Patients were not directly incentivized.

5. Please provide detail on the explanatory variables chosen.

Detail on explanatory variable is now included in Supporting Information document under heading “Explanatory Variable Operational Definitions”

6. Please provide citations for the literature review of the explanatory variables.

16.	Hernandez AF, Greiner MA, Fonarow GC, Hammill BG, Heidenreich PA, Yancy CW, et al. Relationship between early physician follow-up and 30-day readmission among Medicare beneficiaries hospitalized for heart failure. JAMA. 2010;303: 1716–1722. doi:10.1001/jama.2010.533

17.	Lee KK, Yang J, Hernandez AF, Steimle AE, Go AS. Post-discharge Follow-up Characteristics Associated With 30-Day Readmission After Heart Failure Hospitalization. Med Care. 2016;54: 365–372. doi:10.1097/MLR.0000000000000492

18.	Tung Y-C, Chang G-M, Chang H-Y, Yu T-H. Relationship between Early Physician Follow-Up and 30-Day Readmission after Acute Myocardial Infarction and Heart Failure. PLoS One. 2017;12: e0170061. doi:10.1371/journal.pone.0170061

21.	Hess CN, Shah BR, Peng SA, Thomas L, Roe MT, Peterson ED. Association of early physician follow-up and 30-day readmission after non-ST-segment-elevation myocardial infarction among older patients. Circulation. 2013;128: 1206–1213. doi:10.1161/CIRCULATIONAHA.113.004569

7. It is unclear why the authors chose to make Medicaid binary. Were participants who did not have insurance included in this population? If so I would encourage the author to recategorize insurance into multiple types (Medicaid, private, none) as this could have an impact of follow up.

The main interest was to examine whether patients on Medicaid had lower rates of follow-up compared to patients on Medicare or private insurance. Study sample had 51 (0.79%) patients with ‘No insurance/Self Pay’ and these patients were coded 0 on the binary Medicare measure.

8. How were hospital level characteristics chosen as controls? Please provide citations.

While we have named the variable on the presence of any transitional care activity a “hospital-level characteristic” (see: Methods section on explanatory variables), we did not include any other hospital-level variables. This is because the IMPACT study did not collect data on hospital-level characteristics. We did not use hospital-level characteristics as controls in models for both study outcomes, post discharge ED visits and scheduling of 7-day follow-up. Of note, we included an indicator variable for each hospital to account for hospital-level heterogeneity, we also used a hospital-level interaction term of Black race and site for an analysis on Black race and early follow-up at a hospital level.

9. Please provide more information on the Charlson score. What is included in it and how is it scored? Please provide a citation for it as well.

Charlson score was calculated according to methodology described in Charson et al (1987). Nineteen conditions used in calculating Charlson score were: Tumor without metastasis (exclude if >5 years from diagnosis), Leukemia (acute or chronic), Lymphoma Metastatic solid tumor, Myocardial infarction (history, not ECG changes only) Congestive heart failure (CHF), Diabetes without end-organ damage (excludes diet-controlled alone), Diabetes with end-organ damage (retinopathy, neuropathy, nephropathy, or brittle diabetes), Mild liver disease (without portal hypertension, includes chronic hepatitis) Moderate or severe liver disease, Cerebrovascular disease (CVA with mild or no residual or TIA) Hemiplegia, Dementia, AIDS (not just HIV positive) Chronic pulmonary disease Connective tissue disease Moderate or severe renal disease Peptic ulcer disease, Peripheral vascular disease (includes aortic aneurysm >= 6 cm).

Article that developed the Index and one that used it for predicting readmission:

Charlson, M. E., Pompei, P., Ales, K. L., & MacKenzie, C. R. (1987). A new method of classifying prognostic comorbidity in longitudinal studies: development and validation. Journal of chronic diseases, 40(5), 373-383.

Buhr, R. G., Jackson, N. J., Kominski, G. F., Dubinett, S. M., Ong, M. K., & Mangione, C. M. (2019). Comorbidity and thirty-day hospital readmission odds in chronic obstructive pulmonary disease: a comparison of the Charlson and

These details are provided in: ED Disparities Paper_Statistical Analysis Section.docx

11. Please clarify how the association between early follow up and ED returns was statistically determined. This is missing from the methods section.

The model is clarified by the following addition to methods “Adjusted risk ratios (aRR) were estimated from multivariable logistic regressions evaluating the association between the main outcomes and explanatory variables, with the addition of early follow-up as an explanatory variable in the outcome of ED returns. “

12. Please clarify how zipcode-based neighborhood income is constructed.

These details are provided in Supplement: “**Neighborhood income** is zip code median household income from the 2006-2010 American Community Survey (<https://www.psc.isr.umich.edu/dis/census/Features/tract2zip/>), and was measured in units of $1000 for purposes of analysis .

RESULTS
1. Please clarify in Table 1 what ($1000) means for neighborhood income –

This has been added to the footnotes of the table under caption a: Neighborhood income is zip code median household income measured in units of $1K

2. The author should consider creating a second Table 1 split by Race in the supplementary materials.

This is now in Supplement Table stratified by race, and added to the results “(and stratified by race in S1 Table)“

3. Please provide a table for the adjusted analysis of early follow up visits.

This is now added to supplement as S3 table of adjusted analysis of early follow up visit and referenced in results.

4. The authors label the variables as “predictors” in Figure 1. However, this is not a predictive model. Please change to “variables”– Panel A: predictor changed to Variables; Panel B: predictor changes to Site; and both plots saved as png.
5. Please make Figure 1 bigger.

The terms have been changed to “variables” and “site” PLOS ONE formatting guidelines instructs to safe as TIFF, which we have now done <https://journals.plos.org/plosone/s/file?id=wjVg/PLOSOne_formatting_sample_main_body.pdf>

DISCUSSION
1. Please provide detail as to what the author means by “although Black patients have worse HF outcomes”.

This is now explained by the first sentence*: “Black patients face the worst heart disease survival rates of all racial groups[1] and in particular, black patients experience higher age-adjusted heart failure (HF) death rates compared to White patients.[“*

2. Please provide citations for the sociological variables mentioned on line 186.

This has been updated to state the variables used in the already cited Roberts [citation 30] study “such as educational attainment, poverty and social isolation.”

3. Please provide strengths of the study.

This is now addressed in the discussion*: “Study strength include: incorporation of detailed patient-level and visit-level variables to help understand contextual factors of healthcare utilization, incorporation of high-quality chart abstracted all-payer data across a wide variety of hospital situated in geographic areas with diverse demographics.”*

4. Please provide policy recommendations or implications of the study.

This is now addressed in the discussion as a call to investigate other national quality measures for inequity outcomes and potentially proposed transitional care measures as a process-based alternative, and the importance to measure equity of effect of measures : “ *Investigations into transitional care measures that may improve healthcare access is increasingly important given the potential negative effect of influential national policies that may not adequately take into consideration sociodemographic and community factors. [31,32] One such example is with the Centers for Medicare and Medicaid Services (CMS)’s Hospital Readmissions Reduction Program (HRRP), a program which does not incorporate social determinants [33,34] but penalizes hospitals for higher than expected rates of readmission. The policy implications of our findings highlight the need to measure, monitor and mitigate the potential inequitable effects of national quality measures, especially ones that could exacerbate financial challenges for already struggling safety-net hospitals. Incorporating modifiable, process-based measures, such as equity in offering of transitional care services, may represent a future model for federal quality measure consideration. “*

5. Please provide an additional proofread of the manuscript.

Have now done so, thank you for your detailed edits and suggestions!

Reviewer #2: Overall, I think this paper is shining light on an important issue of inequitable interventions related to heart failure care. My largest critique is that nowhere is the cause of these findings named. There is a rather bland mention of "bias" in the discussion. In my view, what this paper is describing is institutionalized racism, and I believe that should be explicitly stated in this paper. There is plenty of great work to cite and source this discussion from, but perhaps two papers that I might recommend integrating are: (Churchwell et al., 2020; Elias & Paradies, 2021)
Related to this comment, the authors do not justify excluding those outside of the self-reported White and Black race categories. There is one comment that the variables chosen were based on theoretical understanding of risk factors for early emergency department returns, which is conceptually sound. While I am not suggesting that the analysis should have included all self-reported races and ethnicities, I think the authors need to justify this choice, as it may have been found that there is also inequitable care of other racial groups.

Thank you for these citations. The introduction has been expanded to provide further justification for focusing on black patients- that they have the worst survival rates of all races and there is evidence of a difference in care, and that this difference may be due to bias creates a narrative that there are specific clinical management changes that can be altered to potentially improve the overall health outcomes for black patients.

Next, the introduction does a better job of differentiating, but in certain places in the paper (such as on line 110) it seems as though the authors include Black race with other markers of clinical vulnerability. Although genetics certainly play a role in heart failure risk for black people (Nayak et al., 2020), what this paper is describing is access to health services rather than genetic risk of HF. If the authors are meaning to talk about genetic risk of HF that should be explicitly stated. I would argue that being Black does not make a person inherently clinically vulnerable. While genetics may predispose a person to some conditions, I do not think that is the phenomenon this paper is focusing on. This needs to be carefully addressed by authors throughout the paper.

We have updated discussion to frame race as marker of social structure impact rather than a marker of genetic predisposition.

Finally, in reading the figures, some interesting protective factors (such as PCP identified in discharge summary or being married) aren't discussed very much- depending on word count, the authors may want to consider adding some text to describe those more in detail.

We have already added significantly to the word count to discuss race, so unfortunately we will not be able to cover further discussion on this.


Minor feedback

• Line 97- three charts? Is this a typo? This seems very low to me but if not please ignore.

We have double checked this and not been told otherwise.

• Line 101: Otherwise in the paper heart failure is HF but here it is spelled out, is this intentional?

Updated to HF.

References

Churchwell, K., Elkind, M. S. V., Benjamin, R. M., Carson, A. P., Chang, E. K., Lawrence, W., Mills, A., Odom, T. M., Rodriguez, C. J., Rodriguez, F., Sanchez, E., Sharrief, A. Z., Sims, M., Williams, O., & On behalf of the American Heart Association. (2020). Call to Action: Structural Racism as a Fundamental Driver of Health Disparities: A Presidential Advisory From the American Heart Association. Circulation, 142(24). <https://doi.org/10.1161/CIR.0000000000000936>

Elias, A., & Paradies, Y. (2021). The Costs of Institutional Racism and its Ethical Implications for Healthcare. Journal of Bioethical Inquiry, 18(1), 45–58. <https://doi.org/10.1007/s11673-020-10073-0>

Nayak, A., Hicks, A. J., & Morris, A. A. (2020). Understanding the Complexity of Heart Failure Risk and Treatment in Black Patients. Circulation: Heart Failure, 13(8). <https://doi.org/10.1161/CIRCHEARTFAILURE.120.007264>

6. PLOS authors have the option to publish the peer review history of their article ([what does this mean?](https://journals.plos.org/plosone/s/editorial-and-peer-review-process#loc-peer-review-history)). If published, this will include your full peer review and any attached files.


If you choose “no”, your identity will remain anonymous but your review may still be made public.


**Do you want your identity to be public for this peer review?** For information about this choice, including consent withdrawal, please see our [Privacy Policy](https://www.plos.org/privacy-policy).

Reviewer #1: **Yes:** Hallie Dau

Reviewer #2: **Yes:** Alexandra Lukey

[NOTE: If reviewer comments were submitted as an attachment file, they will be attached to this email and accessible via the submission site. Please log into your account, locate the manuscript record, and check for the action link "View Attachments". If this link does not appear, there are no attachment files.]

While revising your submission, please upload your figure files to the Preflight Analysis and Conversion Engine (PACE) digital diagnostic tool, <https://pacev2.apexcovantage.com/>. PACE helps ensure that figures meet PLOS requirements. To use PACE, you must first register as a user. Registration is free. Then, login and navigate to the UPLOAD tab, where you will find detailed instructions on how to use the tool. If you encounter any issues or have any questions when using PACE, please email PLOS at [figures@plos.org](mailto:figures@plos.org). Please note that Supporting Information files do not need this step.
