# Supplementary material for: Emergency department returns and early follow-up visits after heart failure hospitalization: Cohort study examining the role of race
Source: PLoS One. 2022 Dec 22;17(12):e0279394. doi: 10.1371/journal.pone.0279394 (PMC9778499; doi:10.1371/journal.pone.0279394)
Supplement: Attachment — Submitted filename: Response to reviewers_10.31.22.docx [file pone.0279394.s007.docx]

From: **PLOS ONE** <[em@editorialmanager.com](mailto:em@editorialmanager.com)>
Date: Thu, 13 Oct 2022 at 10:09
Subject: PLOS ONE Decision: Revision required [PONE-D-21-38345R1] - [EMID:cbb389cf884c6d73]
To: Rachel Emily Solnick <[rachel.solnick@gmail.com](mailto:rachel.solnick@gmail.com)>

PONE-D-21-38345R1

Examination of Racial Disparities in Early Follow-up Visits and ED Returns after Heart Failure Hospitalization

PLOS ONE

Dear Dr. Solnick,

Thank you for submitting your manuscript to PLOS ONE. After careful consideration, we feel that it has merit but does not fully meet PLOS ONE’s publication criteria as it currently stands. Therefore, we invite you to submit a revised version of the manuscript that addresses the points raised during the review process.

Editor's comments:

How did the authors calculate risk ratios when they used the logistic regression analysis?

The method used in the manuscript for calculating aRRs following estimation of a logistic model is describe in Norton et al: Norton EC, Miller MM, Kleinman LC. Computing Adjusted Risk Ratios and Risk Differences in Stata. *Stata J*. 2013;13(3):492-509. doi:10.1177/1536867X1301300304

I agree with the reviewer #1 - the paper is misaligned starting from the title to the selected analytical approach. If the authors are interested in investigating the relationship between race and early follow-up, then a separate model should be built for this purpose (usually, covariates included in the model should be selected based on a solid rationale and literature review – a directed acyclic graph). The second question, if the relationship between Medicaid and early follow-up is investigated, there needs to be another separate model built (again, covariates selection should be based on epidemiological/clinical understanding of causal relationships with other covariates). If the research question is to describe factors associated with early follow-up, then it should be explicitly stated in the research aim and appropriate analysis should be selected without necessary emphasizing that the relationship between race and the outcome is unbiased and the “true” association is derived from this model – as an exploratory models are not designed to investigate each variable independently.

The purpose of the scheduled follow-up model was not to explore factors associated with this outcome, but to assess the relationship between Black race and scheduled follow-up adjusting for potential confounders. The Statistical Analysis section was revised to make this point clearer. Potential confounders used in model estimation were based on prior literature as cited and were expected to be correlated with race and with the scheduled follow-up.

However, it should be noted that in the model selection process more emphasis was given to certain confounders. For example, we retained the four LACE risk scoring items in the model regardless of statistical significance, as LACE Instrument is designed for predicting patient risk of mortality and post discharge hospital utilization. ( Based on the work by Van Walraven C, Dhalla IA, Bell C, Etchells E, Stiell IG, Zarnke K, Austin PC, Forster AJ. 2010. Derivation and validation of an index to predict early death or unplanned readmission after discharge from hospital to the community. CMAJ: Canadian Medical Association Journal 182(6):551557 DOI 10.1503/cmaj.091117.)

And I could not find the results for the secondary aim investigating the relationship between early follow-up and 30-day ED return.

The outcome of ED return has now been highlighted with a new subtitle in the second paragraph of the results section. Logistic model results for relationship between early follow-up and 30-day ED return that were in S1-Fig is now provided in regular Figure 1.

The relationship between early follow-up and 30-day ED return is in fact the first aim of the study, and investigating racial disparities in scheduled follow-up is the second aim. The manuscript is revised to reflect this order. The Statistical Analysis section was revised to explain more clearly the analysis and model estimation. With these revisions we hope to clarify that two logistic models were estimated: (1) a model to evaluate the relationship between ED returns and early follow-up, (2) a model to evaluate the association between Black race and early follow-up (now Fig 2a); and related model stratified by hospital site (now Fig 2b)

Please submit your revised manuscript by Nov 27 2022 11:59PM. If you will need more time than this to complete your revisions, please reply to this message or contact the journal office at [plosone@plos.org](mailto:plosone@plos.org). When you're ready to submit your revision, log on to <https://www.editorialmanager.com/pone/> and select the 'Submissions Needing Revision' folder to locate your manuscript file.

Please include the following items when submitting your revised manuscript:

- A rebuttal letter that responds to each point raised by the academic editor and reviewer(s). You should upload this letter as a separate file labeled 'Response to Reviewers'.
- A marked-up copy of your manuscript that highlights changes made to the original version. You should upload this as a separate file labeled 'Revised Manuscript with Track Changes'.
- An unmarked version of your revised paper without tracked changes. You should upload this as a separate file labeled 'Manuscript'.

If you would like to make changes to your financial disclosure, please include your updated statement in your cover letter. Guidelines for resubmitting your figure files are available below the reviewer comments at the end of this letter.

If applicable, we recommend that you deposit your laboratory protocols in [protocols.io](http://protocols.io) to enhance the reproducibility of your results. Protocols.io assigns your protocol its own identifier (DOI) so that it can be cited independently in the future. For instructions see: <https://journals.plos.org/plosone/s/submission-guidelines#loc-laboratory-protocols>. Additionally, PLOS ONE offers an option for publishing peer-reviewed Lab Protocol articles, which describe protocols hosted on [protocols.io](http://protocols.io). Read more information on sharing protocols at <https://plos.org/protocols?utm_medium=editorial-email&utm_source=authorletters&utm_campaign=protocols>.

We look forward to receiving your revised manuscript.

Kind regards,

Alpamys Issanov
Academic Editor
PLOS ONE

[Note: HTML markup is below. Please do not edit.]

Reviewers' comments:

Reviewer's Responses to Questions

**Comments to the Author**

1. If the authors have adequately addressed your comments raised in a previous round of review and you feel that this manuscript is now acceptable for publication, you may indicate that here to bypass the “Comments to the Author” section, enter your conflict of interest statement in the “Confidential to Editor” section, and submit your "Accept" recommendation.

Reviewer #1: (No Response)

Reviewer #2: All comments have been addressed

2. Is the manuscript technically sound, and do the data support the conclusions?

The manuscript must describe a technically sound piece of scientific research with data that supports the conclusions. Experiments must have been conducted rigorously, with appropriate controls, replication, and sample sizes. The conclusions must be drawn appropriately based on the data presented.

Reviewer #1: No

Reviewer #2: (No Response)

3. Has the statistical analysis been performed appropriately and rigorously?

Reviewer #1: No

Reviewer #2: (No Response)

4. Have the authors made all data underlying the findings in their manuscript fully available?

The [PLOS Data policy](http://www.plosone.org/static/policies.action#sharing) requires authors to make all data underlying the findings described in their manuscript fully available without restriction, with rare exception (please refer to the Data Availability Statement in the manuscript PDF file). The data should be provided as part of the manuscript or its supporting information, or deposited to a public repository. For example, in addition to summary statistics, the data points behind means, medians and variance measures should be available. If there are restrictions on publicly sharing data—e.g. participant privacy or use of data from a third party—those must be specified.

Reviewer #1: No

Reviewer #2: (No Response)

5. Is the manuscript presented in an intelligible fashion and written in standard English?

PLOS ONE does not copyedit accepted manuscripts, so the language in submitted articles must be clear, correct, and unambiguous. Any typographical or grammatical errors should be corrected at revision, so please note any specific errors here.

Reviewer #1: Yes

Reviewer #2: (No Response)

6. Review Comments to the Author

Please use the space provided to explain your answers to the questions above. You may also include additional comments for the author, including concerns about dual publication, research ethics, or publication ethics. (Please upload your review as an attachment if it exceeds 20,000 characters)

Reviewer #1: INTRODUCTION

Please provide an additional proofread of the manuscript. There are some grammatical errors. For example, black is not capitalized on line 86

Thank you for your review, we have conducted an additional proofread of the manuscript, and corrected this error above.

Lines 117 and 118 should connect to be one paragraph.

This has been corrected.

This focus of this paper is misaligned. The title of the paper indicates that the focus is on racial disparities. However, the objective seems to focus on Black race and Medicaid as a marker of low-income status. Please clarify and realign.

Racial disparities (between Black and White race) in the offering of early follow-up visits is one of the focuses of the paper, in addition to the association between early follow-up and emergency department returns. Medicaid/ low-income is not the focus of the paper. We will remove Medicaid from the Introduction.

Low-income status is mentioned in the objective as one of the focuses of the paper. However, it is not discussed in the introduction. Rather the introduction solely focuses on race. Please clarify.

The objective is analyzing the association between early follow-up and emergency department returns, and of race and early follow up. Low-income status has been removed from the objective.

METHODS

Is the missing data range on 143 a typo?

Changed text to: Missing data ranged from 0% to 1.89%

The introduction states that the aim is to examine if Black race and Medicaid are associated with a scheduled 7 day follow up. However, in the methods section numerous explanatory variables are mentioned (demographics, Charlston score, patient hospital characteristics). Many of these seem to be potential confounders rather than explanatory variables (if so, the aim of the study should be changed). Please clarify as this is a major concern.

Medicaid is considered as a confounder, not an explanatory variable, and the manuscript has been changed accordingly. As described above, apart from the variables of interest (which are described in the revised Statistical Analysis section) the remaining factors including four LACE risk scoring items, prior hospital admissions, other clinical and demographic variables were considered as confounders and not as explanatory variables. For the outcome ED returns the variable of interest (explanatory variable) was scheduled follow-up. For the outcome scheduled follow-up the variable of interest was race.

Please provide an explanation of the sensitivity analysis conducted. The primary analysis uses aRR. However the sensitivity analysis seems to use aOR. Details are needed in the methods section of how this analysis was constructed.

Sensitivity analysis was conducted to see whether findings were sensitive to exclusion of patients discharged during the Covid pandemic. Sensitivity analysis was conducted by re-fitting the main regression models using a sample that excluded patients discharged from April 1, 2020 through the end of sample data on September 30, 2020.

For consistency, the sensitivity analysis is now in aRR not aOR in the paragraph before ‘Discussion’.

RESULTS

Please review footnote (a) in table 1. It states a linear regression was used for continuous measures. This statistically does not make sense as the data is bivariate. Do the authors possibly mean a t-test?

T-tests were completed for continuous measures and Table 1 footnote text was revised to “… p-values for continuous measures are based on T-tests”.

DISCUSSION

Please provide context of the RCT mentioned on line 294. When and where was it conducted?

This is now described in location and with more detail: “. Our theory—that transitional care can avert ED returns—is supported by previous randomized trials based in Canada, which found that HF patients randomized to transitional care after discharge had a reduction in rate of ED returns [19]: in one study in 2000 by Harrison et al. of 200 patients, nurse-led transitional care of structured home visits and telephone follow-up reduced ED visits; in the second study in 1998 by Tsuyuki et al. of 276 patients, transitional care including patient education and telephone follow-up reduced cardiovascular-related ED visits.”

Reviewer #2: (No Response)

7. PLOS authors have the option to publish the peer review history of their article ([what does this mean?](https://journals.plos.org/plosone/s/editorial-and-peer-review-process#loc-peer-review-history)). If published, this will include your full peer review and any attached files.


If you choose “no”, your identity will remain anonymous but your review may still be made public.


**Do you want your identity to be public for this peer review?** For information about this choice, including consent withdrawal, please see our [Privacy Policy](https://www.plos.org/privacy-policy).

Reviewer #1: **Yes:** Hallie Dau

Reviewer #2: **Yes:** Alexandra Luke
